# A P2X receptor from the tardigrade species *Hypsibius dujardini *with fast kinetics and sensitivity to zinc and copper

**DOI:** 10.1186/1471-2148-9-17

**Published:** 2009-01-20

**Authors:** Selvan Bavan, Volko A Straub, Mark L Blaxter, Steven J Ennion

**Affiliations:** 1 Department of Cell Physiology and Pharmacology, University of Leicester, PO Box 138, Leicester, LE1 9HN, UK; 2 Institute of Evolutionary Biology, The Ashworth laboratories, University of Edinburgh, Edinburgh, UK

## Abstract

**Background:**

Orthologs of the vertebrate ATP gated P2X channels have been identified in *Dictyostelium *and green algae, demonstrating that the emergence of ionotropic purinergic signalling was an early event in eukaryotic evolution. However, the genomes of a number of animals including *Drosophila melanogaster *and *Caenorhabditis elegans*, both members of the Ecdysozoa superphylum, lack P2X-like proteins, whilst other species such as the flatworm *Schistosoma mansoni *have P2X proteins making it unclear as to what stages in evolution P2X receptors were lost. Here we describe the functional characterisation of a P2X receptor (*Hd*P2X) from the tardigrade *Hypsibius dujardini *demonstrating that purinergic signalling is preserved in some ecdysozoa.

**Results:**

ATP (EC_50 _~44.5 μM) evoked transient inward currents in *Hd*P2X with millisecond rates of activation and desensitisation. *Hd*P2X is antagonised by pyridoxal-phosphate-6-azophenyl-2',4' disulfonic acid (IC_50 _15.0 μM) and suramin (IC_50 _22.6 μM) and zinc and copper inhibit ATP-evoked currents with IC_50 _values of 62.8 μM and 19.9 μM respectively. Site-directed mutagenesis showed that unlike vertebrate P2X receptors, extracellular histidines do not play a major role in coordinating metal binding in *Hd*P2X. However, H306 was identified as playing a minor role in the actions of copper but not zinc. Ivermectin potentiated responses to ATP with no effect on the rates of current activation or decay.

**Conclusion:**

The presence of a P2X receptor in a tardigrade species suggests that both nematodes and arthropods lost their P2X genes independently, as both traditional and molecular phylogenies place the divergence between Nematoda and Arthropoda before their divergence from Tardigrada. The phylogenetic analysis performed in our study also clearly demonstrates that the emergence of the family of seven P2X channels in human and other mammalian species was a relatively recent evolutionary event that occurred subsequent to the split between vertebrates and invertebrates. Furthermore, several characteristics of *Hd*P2X including fast kinetics with low ATP sensitivity, potentiation by ivermectin in a channel with fast kinetics and distinct copper and zinc binding sites not dependent on histidines make *Hd*P2X a useful model for comparative structure-function studies allowing a better understanding of P2X receptors in higher organisms.

## Background

Vertebrate P2X receptors comprise a family of ligand gated ion channels activated by extracellular ATP [1]. They form homo or heteromeric trimers with each monomer consisting of intracellular amino and carboxy termini, two transmembrane domains and a large glycosylated extracellular region containing five disulphide bonds [2] and the agonist binding site [3]. Mammalian species possess seven distinct P2X subtypes (P2X_1–7_) that play important roles in a wide range of physiological processes including neurotransmission, platelet aggregation, smooth muscle contractility and bone formation [4,5]. Many studies have also described potential roles for ATP as an extracellular signalling molecule in a range of invertebrate phyla [6] and plants [7,8] leading to the assumption that ATP is a primitive signalling molecule and that the emergence of purinergic receptors occurred relatively early in evolution [9]. This assumption is supported by the definitive molecular and functional identification of P2X receptors in the slime mould *Dictyostelium discoideum *[10], the green alga *Ostreococcus tauri *[11] and the choanoflagellate *Monosiga brevicollis *[11]. However, several non-vertebrate organisms for which full genome data are available lack P2X-like genes, including *Anopheles gambiae, Caenorhabditis elegans*, *Drosophila melanogaster*, and *Apis mellifera*. Arthropods and nematodes are members of the protostome superphylum Ecdysozoa. Given that P2X receptors are present in choanoflagellates, believed to be the sister group to the Metazoa, and in some representatives of the protostome superphylum Lophotrochozoa including *Schistosoma mansoni *[12] and *Lymnaea stagnalis *[13], the absence of P2X receptors from the above fully sequenced nematode and arthropod genomes suggests a loss of this class of gene in an ancestor of the Ecdysozoa.

We identified a partial P2X-like sequence in expressed sequence tag (EST) data from the tardigrade *Hypsibius dujardini*. Tardigrades are microscopic animals around 200 to 500 μm in length that inhabit both marine and fresh water habitats [14,15]. They possess a fascinating ability to desiccate into a reversible state of metabolic suspension called cryptobiosis allowing them to survive for many decades in harsh environments, such as lack of oxygen, extremes in temperature, and high pressure, before rehydration to an active state within minutes [16]. Tardigrades are placed in their own phylum, Tardigrada and share features with both arthropods and nematodes. Like arthropods they possess legs and a distinctly segmented body. However they also have a triradiate pharynx more reminiscent of the nematodes. Indeed, molecular phylogenetic analyses support tardigrades as part of the Ecdysozoa [17,18]. Functional confirmation that tardigrades possess P2X receptors would expand our emerging knowledge of P2X phylogeny and better estimate the pattern of loss of P2X in nematodes and arthropods. Furthermore, a better understanding of P2X receptor function in simple invertebrate organisms may help shed new light on structure-function aspects of human P2X receptors in health and disease by virtue of conservation of functionally important amino acid residues between evolutionary remote P2X receptors and the identification of novel P2X mediated signalling pathways. The aim of this present work therefore was to determine whether the *Hypsibius dujardini *EST sequence corresponds to a gene encoding a functional P2X receptor and to determine its pharmacological properties. This was achieved by expression of cRNA encoding the *Hypsibius dujardini *P2X gene in *Xenopus *oocytes to enable two electrode voltage clamp recordings of ATP evoked membrane currents. Using this approach we show that the *Hypsibius dujardini *EST sequence does indeed code a functional P2X receptor with fast activation and desensitisation kinetics and similar to some vertebrate P2X channels, is potentiated by ivermectin and inhibited by zinc and copper.

## Results

### Sequence analysis of HdP2X

**Figure 1:**
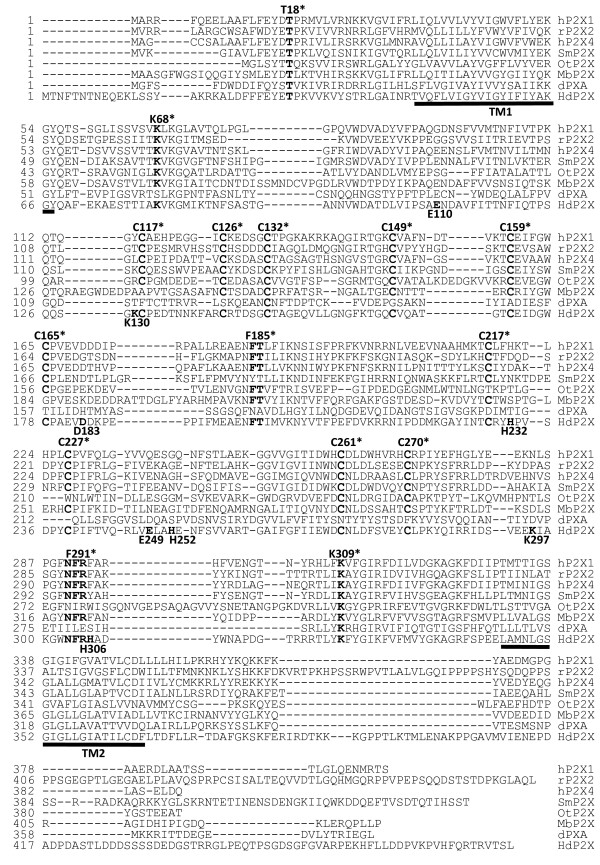
**Amino acid alignment of *Hd*P2X**. The predicted amino acid sequence of *Hd*P2X was aligned with human P2X_1, and 4_, rat P2X_2 _and non vertebrate P2X channels from *S. mansoni *(smP2X) [12]*O. tauri *(OtP2X) [11], *M. brevicollis *(MbP2X) [11] and *D. discoideum *(dP2XA) [10]. Predicted transmembrane regions in *Hd*P2X (TopPred algorithm [19]) are depicted by black horizontal lines. Functionally important amino acid residues using human P2X_1 _residue numbering (indicated by *) are highlighted in bold above the sequence including positively charged lysine residues, FT and NFR motifs, conserved cysteines and a conserved consensus protein kinase C phosphorylation site. Point mutations made in *Hd*P2X to investigate zinc and copper binding are indicated in bold below the sequence.

**Figure 2:**
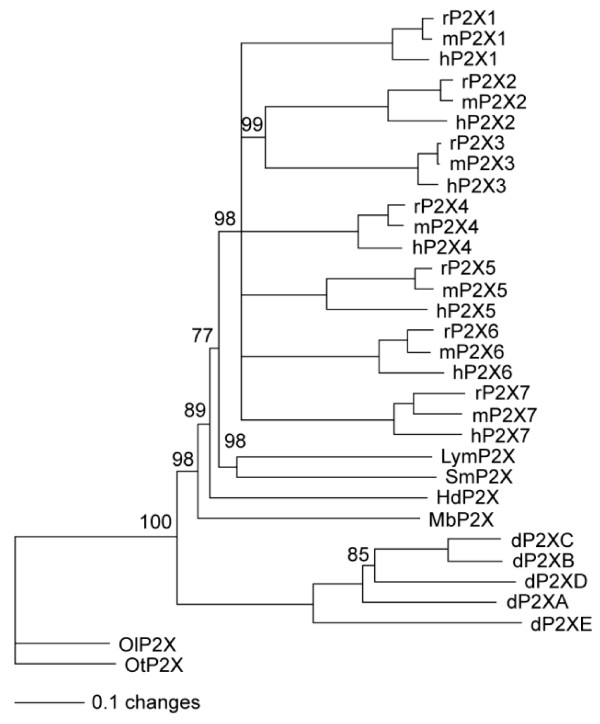
**Phylogenetic relationships of *Hypsibius dujardini *P2X**. Aligned protein sequences from P2X homologues from *H. dujardini *(Hd) (Tardigrada), vertebrates (represented by human (h), mouse (m) and rat (r); these taxa have seven paralogous P2X genes (P2X_1–7_)), *S. mansoni *(Sm) (Platyhelminthes), *L. stagnalis *(Lym) (Mollusca), *M. brevicollis *(Mb) (Choanoflagellida), *D. discoideum *(d) (Mycetozoa; Dictyosteliida; five paralogues) and two *Ostreococcus *species, *O. tauri *(Ot) and *O. lucimarinus *(Ol) (Chlorophyta; Prasinophyceae) were analysed using Neighbour Joining and maximum parsimony methods. Both methods agreed on the well supported nodes figured in this phylogram. Nodes were supported with bootstraps of 100% unless otherwise indicated; all nodes with less than 75% bootstrap support were collapsed.

Existing EST data for clone Hd_mx23_13F10 consisted of 330 bp from the 5' end of a cDNA showing similarity to vertebrate P2X receptors. Sequencing of the full insert of this clone showed that it contained an insert of 1743 bp with an open reading frame of 1440 bp. The nucleotide sequence of this cDNA has been submitted to the GenBank database [EU979525]. The coding sequence of *Hd*P2X is 480 amino acids in length and is predicted to contain intracellular amino and carboxy termini and two transmembrane helices (residues 47 – 67 and 345 – 365) by the TopPred algorithm [19] (Fig. 1). From the human P2X_1–7 _family, *Hd*P2X was most similar to P2X_1_, P2X_3 _and P2X_4 _with sequence identities of 36.1, 36.5 and 38.4% respectively. However, phylogenetic analysis of the *Hd*P2X protein alongside vertebrate and other P2X sequences suggests that, like other invertebrate P2X receptor proteins, *Hd*P2X does not appear to be a member of any of the seven vertebrate P2X receptor subtypes, but instead represents an ortholog to the ancestor of the vertebrate paralog groups (Fig. 2). Sequence identity with the *S. mansoni*, *D. discoideum *(dP2XA), and *O. tauri *P2X channels was 34.2%, 15.6% and 26.0% respectively. *Hd*P2X contains a number of conserved features typical to P2X channels including ten cysteine residues in the extracellular domain [2], a consensus protein kinase C phosphorylation site in the amino terminal [20], and the lysine residues and NFR/FT motifs shown to be involved in agonist binding (Fig. 1) [21].

### ATP evoked currents in HdP2X with fast activation and desensitisation kinetics

**Figure 3:**
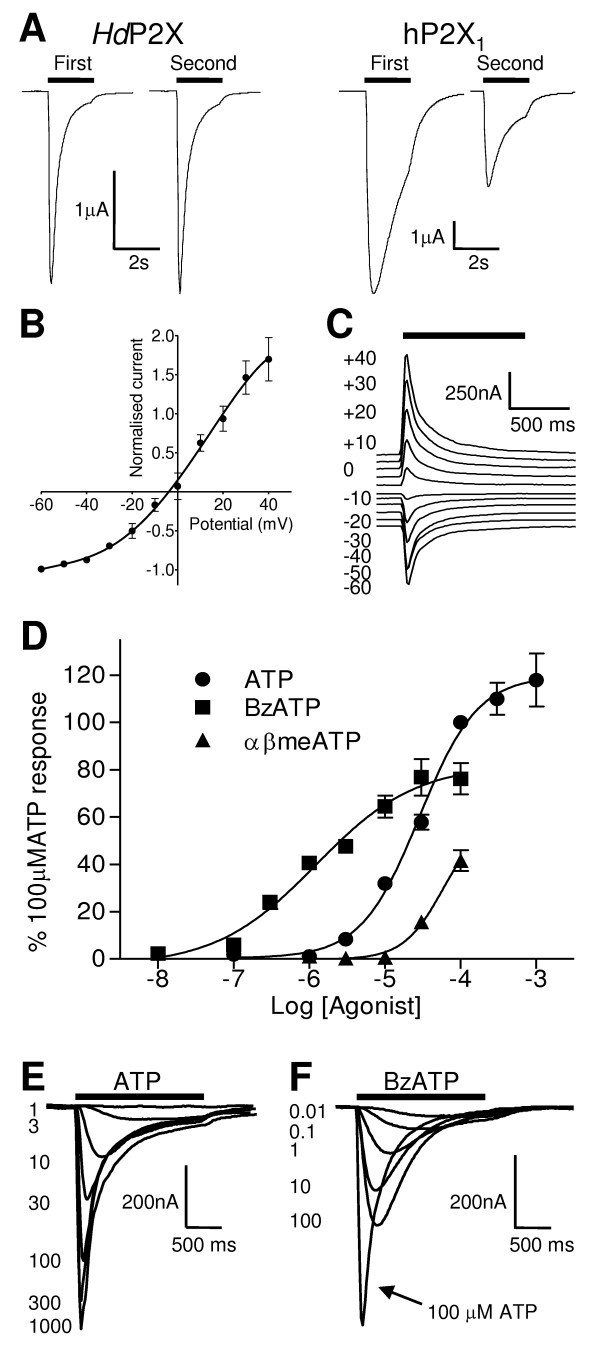
**Properties of ATP evoked currents**. Two-electrode voltage clamp recordings at a holding membrane potential of -60 mV were made from oocytes expressing *Hd*P2X. A. Comparison of *Hd*P2X and human P2X_1 _receptors. Currents were recorded in response to 100 μM ATP, indicated by bar. *Hd*P2X displays faster current rise and decay times than the human P2X_1 _and displays faster recovery from desensitisation between the first and second applications of agonist (applications 5 minutes apart). B. Current voltage relationship of *Hd*P2X. The reversal potential of ATP mediated currents was determined by recording ATP (100 μM, indicated by bar) induced currents at holding potentials ranging from -100 mV to +40 mV with a 5 minute interval between applications. Currents obtained in different oocytes were normalised to the current obtained at -60 mV for each individual cell (n = 6). C. Example currents for the plot depicted in B. D. Concentration response curves for ATP, Bz-ATP and αβ-me-ATP. Mean currents were normalised to the response given by 100 μM ATP (n = 5–7). E and F. Example currents recorded in response to ATP (E) and Bz-ATP (F) (concentrations in μM, agonist application indicated by bar).

In *Hd*P2X expressing *Xenopus *oocytes clamped at -60 mV, ATP evoked transient inward currents that rapidly desensitised during the continued presence of agonist (Fig. 3A). At 100 μM ATP (~EC_90_) the rise time from 10% to 90% peak amplitude was 71.4 ± 2.5 ms and the current decay from peak to 50% peak was 289.9 ± 16.1 ms (n = 39). The decay of the current in the continued presence of 100 μM ATP was best fit with two exponentials consisting of a fast component with a time constant of 122 ± 26 ms and a relative amplitude of 0.68 ± 0.04 and a slower component with a time constant of 627 ± 148 ms and a relative amplitude of 0.32 ± 0.04 (n = 6). As a direct comparison using the same experimental apparatus application of 100 μM ATP at the fast human P2X_1 _receptor gave rise and decay times of 134.5 ± 11.0 ms and 727.0 ± 88.6 (n = 15) ms respectively. Both hP2X_1 _(Fig. 3A) and *S. mansoni *P2X [12] display a marked reduction in current amplitude between the very first and second applications of 100 μM ATP five minutes apart (34.39 ± 5.46% and 45.76 ± 5.10% reductions respectively (n = 15)) before producing stable responses with subsequent applications of agonist at five minute intervals. In contrast, with the same experimental protocol, *Hd*P2X showed a much lower reduction in current amplitude (8.96 ± 5.46% (n = 11)) between first and second applications of ATP (first application mean = -1.77 ± 0.30 μA; second application mean = -1.62 ± 0.29 μA (Fig. 3A) demonstrating that *Hd*P2X recovers more rapidly from desensitisation than hP2X_1 _and *S. mansoni *P2X. The current-voltage relationship for *Hd*P2X was obtained by applying 100 μM ATP at a range of holding potentials between -60 mV to and +40 mV. Typical of a non selective cation channel the reversal potential was -3.8 mV (*n *= 6) and the current voltage relationship showed a slight outward rectification over the range of potentials measured (Fig. 3B and 3C).

### Agonists

ATP induced responses at *Hd*P2X in a concentration dependent manner with an EC_50 _of 44.5 μM (pEC_50 _4.5 ± 0.1, *n *= 7) and a Hill slope of 1.12 ± 0.14 (Fig. 3D and 3E). The ATP analogue Bz-ATP also evoked responses at *Hd*P2X with an EC_50 _of 12.2 μM (pEC_50 _5.9 ± 0.2, n = 5) and a Hill slope of 0.7 ± 0.2. The efficacy of Bz-ATP was lower (p < 0.01) than ATP, with the maximum Bz-ATP response 64.6 ± 5.7% that of the maximum response to ATP (Fig. 3D). Currents evoked by Bz-ATP had a slower rise time and rate of desensitization than equivalent ATP evoked currents (p < 0.01 in both cases). With 100 μM BzATP (maximal response) the time from 10%–90% peak current was 142.3 ± 10.2 ms and the rate of current decay from peak to 50% was 446.4 ± 38.9 ms (n = 7) (Fig. 3F). αβ-methylene ATP (αβmeATP) at 100 μM evoked currents that were less than 50% of those evoked by 100 μM ATP. ADP_hex _(hexokinase treated ADP), adenosine, UDP and UTP at 100 μM failed to elicit currents at *Hd*P2X.

### Antagonists

**Figure 4:**
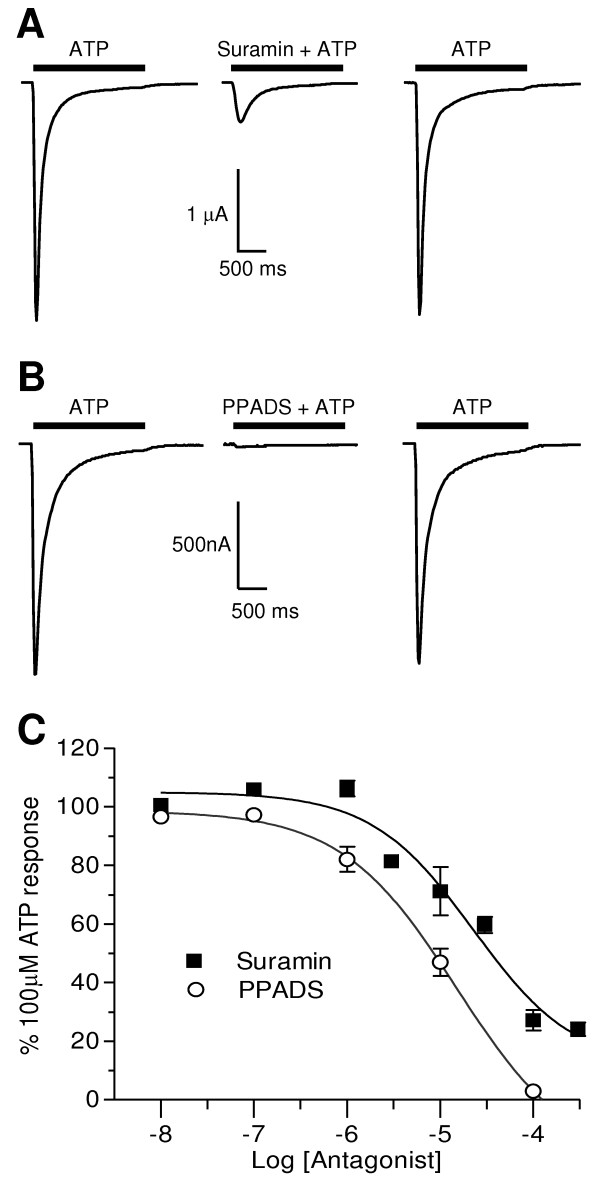
***Hd*P2X is antagonised by suramin and PPADS**. The effects of the P2 receptor antagonists suramin and PPADS were determined in *Xenopus *oocytes expressing *Hd*P2X at a holding membrane potential of -60 mV. A. Example currents in response to 100 μM ATP in the presence and absence of 100 μM suramin (five minutes between sequential applications). B. Example currents in response to 100 μM ATP in the presence and absence of 100 μM PPADS (five minutes between sequential applications). C. Inhibition curves for mean responses to 100 μM ATP in the presence of suramin (closed squares) (n = 5) and PPADS (open circles) (n = 5).

PPADS antagonised ATP evoked currents at *Hd*P2X with an IC_50 _of 15.0 μM (pIC_50 _4.6 ± 0.1, *n *= 5) and a Hill slope of -1.0 ± 0.3 for responses evoked by 100 μM ATP (Fig. 4). Suramin also antagonised *Hd*P2X currents in a concentration dependent manner but not as strongly as PPADS. Suramin inhibited 100 μM ATP responses with an IC_50 _of 22.6 μM (pIC_50 _4.7 ± 0.3, *n *= 5–7) and a Hill slope of -0.8 ± 0.3. A component of the *Hd*P2X current was resistant to suramin and at the highest concentration tested (300 μM), only ~75% of the 100 μM ATP evoked response was inhibited (Fig. 4).

### HdP2X is inhibited by zinc and copper

**Figure 5:**
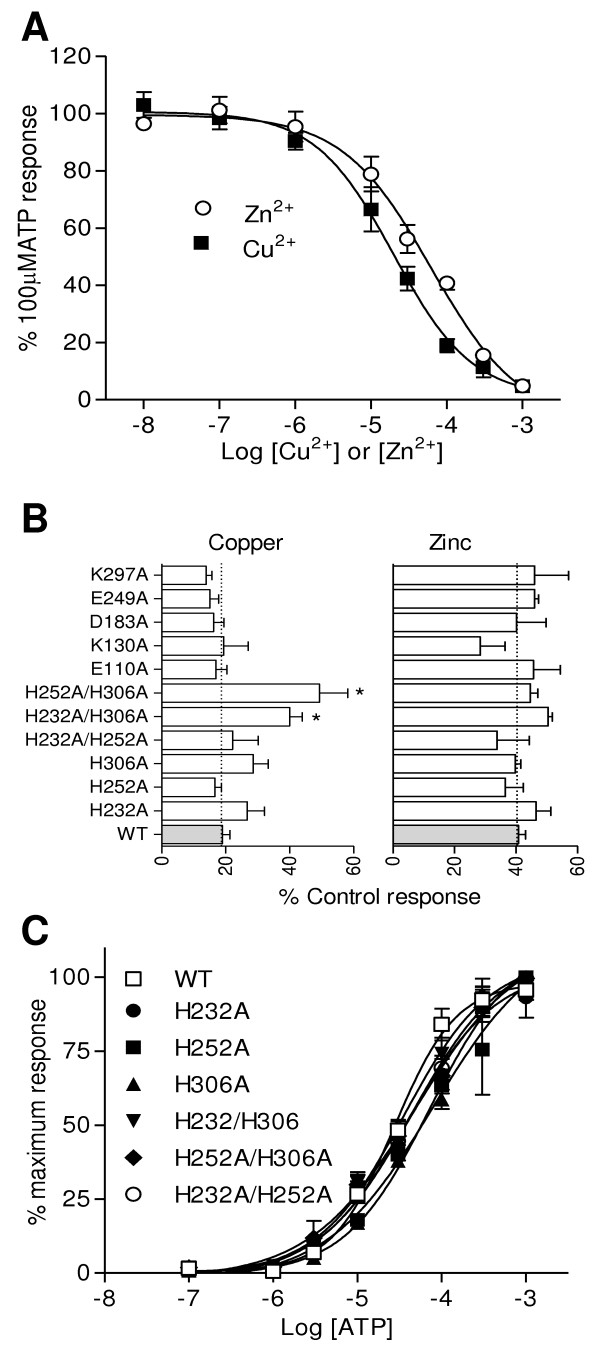
***Hd*P2X receptor currents are inhibited by zinc and copper**. Two-electrode voltage clamp recordings were made from *Xenopus *oocytes expressing *Hd*P2X at a holding potential of -60 mV. A. Concentration response curves for 100 μM ATP (~EC_80_) in the presence of varying concentrations of zinc (open circles) (n = 6) and copper (closed squares) (n = 6). B. Effects of single and double point mutations on inhibition by 100 μM zinc and copper. Mean currents are normalised to the 100 μM ATP response in the absence of zinc or copper for wild type and mutant receptors (n = 7). C. ATP concentration response curves for histidine point mutations (n = 7). For each single and double histidine mutation no significant change in ATP sensitivity was observed suggesting that mutation had not resulted in gross changes in receptor conformation. The triple histidine mutation H232A/H252A/H306A produced a non functional channel and so could not be studied.

Vertebrate P2X receptors can be subject to allosteric modulation by both metal ions and pH [22]. Unlike some vertebrate P2X receptors [23], neither the ATP concentration response curve nor the amplitude of ATP evoked currents for *Hd*P2X were affected by acidic pH (pH6.5) (n = 5, data not shown). The divalent cations zinc and copper however both caused concentration dependent inhibition of ATP-evoked currents (Fig. 5). Zinc inhibited 100 μM ATP currents, with an IC_50 _of 62.8 μM (pIC_50 _4.2 ± 0.14, n = 5–6) and a Hill slope of -0.7 ± 0.1. Copper also acted as an inhibitor of 100 μM ATP currents with an IC_50 _of 19.9 μM (pIC_50 _4.7 ± 0.06, n = 5–6) and a Hill slope of -0.8 ± 0.1. (Fig. 5A). Histidine residues have previously been reported to be involved in zinc and copper inhibition of the P2X_7 _receptor [24,25] and also in the potentiation of P2X_2 _receptor currents by zinc [26]. Three histidine residues are present in the extracellular region of *Hd*P2X and we therefore mutated these residues to alanine both individually and in combination with each other in order to probe their potential involvement in metal ion binding. Using 100 μM zinc as a test concentration, wild type currents evoked by 100 μM ATP (~EC_80_) were inhibited by 59.3 ± 2.2%. The inhibition observed for the single histidine mutants H232A, H252A and H306A and the double histidine mutants H232A/H252A, H252A/H306A and H232A/H306A was not significantly different from wild type (Fig. 5B). The triple histidine mutant H232A/H252A/H306A produced a non functional channel and therefore could not be studied. With copper (100 μM) as the inhibitor, wild type currents were inhibited by 81.0 ± 2.3%. Similar to zinc, the inhibition observed in the single histidine and the H232A/H252A double mutant with copper did not significantly differ from wild type (Fig. 5B). The inhibition observed with the double mutants H232A/H306A and H252A/H306A however was less than wild type (p < 0.05) with H232A/H306A currents inhibited by 60.0 ± 3.9% and H252A/H306A currents inhibited by 50.7 ± 8.9%. In order to assess whether mutation had resulted in a gross change in channel function, the potency of ATP for each histidine mutation was assessed (Fig. 5C) and none of the single or double mutations produced a concentration response curve that differed significantly from wild type. Lysine, aspartic acid, and glutamic acid residues are also potential candidates for coordinating metal binding and a glutamic acid residue in rat P2X_7 _has been shown to be involved in zinc and copper binding [25]. We therefore mutated lysine and negatively charged residues in *Hd*P2X that were specific between *Hd*P2X and *S. mansoni *P2X since this channel is also sensitive to metal ions [27]. Five alanine substitution mutations were created (K297A, E249A, D183A, K130A and E110A) and tested for sensitivity to zinc and copper inhibition (Fig. 5B). No significant difference from the wild type inhibition for either zinc or copper was observed in any of these charged amino acid mutations.

### HdP2X currents are potentiated by Ivermectin

**Figure 6:**
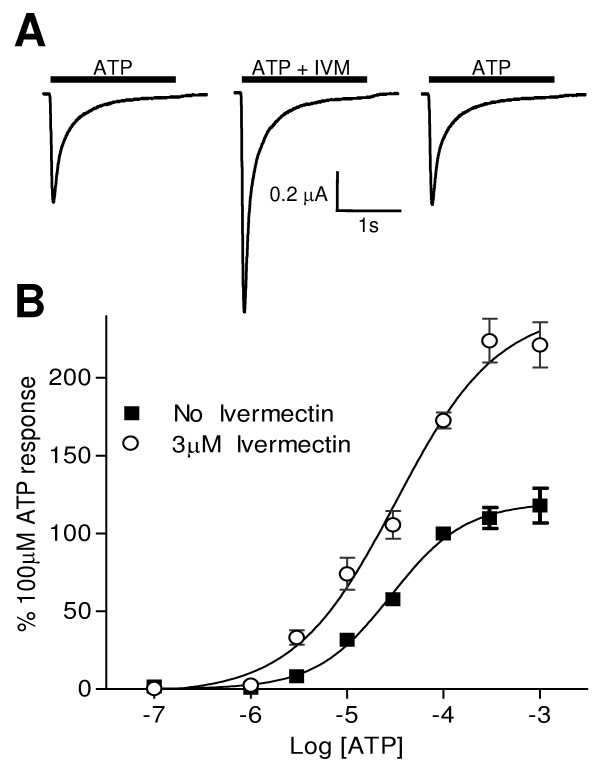
**Ivermectin potentiates ATP evoked currents at *Hd*P2X**. Two-electrode voltage clamp recordings were made from *Xenopus *oocytes expressing *Hd*P2X. A. Representative ATP (100 μM) evoked currents recorded from the same cell in the presence and absence of 3 μM ivermectin (ATP/ivermectin application indicated by bars). Five minutes recovery was allowed between applications and ivermectin was bath perfused at 3 μM in the five minutes preceding the second recording as well as being present in the ATP application. B. Concentration response curves for ATP in the presence (open circles) and absence (closed squares) of 3 μM ivermectin (n = 7). Mean currents were normalised to the response produced by 100 μM ATP in the absence of ivermectin.

Ivermectin, a broad spectrum anti-parasitic agent from the bacterium *Streptomyces avermitilis *is known to potentiate ATP-evoked currents at human and mouse P2X_4 _[28,29] and the *S. mansoni *[12] P2X receptors. We therefore studied the effects of this allosteric modulator on the *Hd*P2X receptor. Ivermectin alone was unable to activate the receptor (data not shown). However, 3 μM ivermectin potentiated the response to a maximal concentration of ATP (300 μM) by 223.8 ± 14.1% of control responses, with no notable effect on the rates of current activation or decay (Fig. 6). Currents were significantly (p < 0.01) potentiated in the presence of 3 μM ivermectin at all concentrations of ATP investigated and the concentration response curve for ATP in the presence of 3 μM ivermectin had an EC_50 _of 32.1 μM (pEC_50 _4.5 ± 0.1, *n *= 5–10) and a Hill slope of 0.8 ± 0.2.

## Discussion

Despite their absence in several key model organisms such as *Saccharomyces cerevisiae*, *Drosophila melanogaster *and *Caenorhabditis elegans*, functional P2X receptors have now been described in a wide range of non-vertebrate metazoan phyla including platyhelminthes [12,27], and molluscs (41), and in representatives of other eukaryotic kingdoms such as dictyostelida [10], prasinophyceae [11] and choanoflagellates [11]. Additional partial and predicted P2X-like proteins can be identified in EST and partial genome data from other animals, including additional molluscs, echinoderms [30] and cnidaria. These data provide an emerging phylogenetic understanding of the evolution of ionotropic purinergic signalling. Alignment of P2X proteins from a diverse range of taxa with the mammalian P2X_(1–7) _families (Fig. 2) clearly shows that the mammalian P2X receptors are a lineage-specific gene expansion, with ortholog triplets evident in mouse, human and rat. A lineage-specific expansion is also evident in *D. discoideum*. In the other taxa, the single P2X receptors identified appear to be orthologous to the entire vertebrate expansion. This strongly suggests that the emergence of a family of seven mammalian P2X receptors was a relatively recent evolutionary event that occurred subsequent to the split between vertebrates and invertebrates. The functional confirmation that P2X receptors exist in the tardigrade *H. dujardini *strongly suggests that the loss of P2X receptors in some nematode (represented by *C. elegans*) and arthropod (represented by the sequenced hexapod genomes) species occurred independently, as both traditional and molecular phylogenies place the divergence between Nematoda and Arthropoda before their divergence from Tardigrada. Pharmacological evidence for purinergic signalling in some nematode and arthropod species [6] suggests that P2X-like receptors may have been maintained in some members of these phyla. Indeed a partial transcript for a P2X gene from the nematode *Xiphinema index *[31] suggests that the loss of P2X in *C. elegans *may have occurred relatively late in nematode evolution, and P2X-like ESTs from the chelicerate *Boophilus microplus *similarly suggest that the loss of P2X receptors in arthropods may be restricted to Hexapoda.

Based on their kinetic parameters and ligand sensitivity, mammalian P2X receptors can be divided into three groups [32]. Group 1 consists of P2X_1 _and P2X_3 _receptors that have high sensitivity for ATP (EC_50 _~1–3 μM) and show rapid channel activation and desensitisation during the continued presence of agonist. Group 2 includes P2X_2_, P2X_4_, P2X_5_, and P2X_6 _receptors with a lower sensitivity for ATP (EC_50 _~10 μM) and much slower desensitization whilst group 3 is represented by P2X_7 _which has a very low sensitivity for ATP (EC _50 _~300 μM) and shows little desensitization. *Hd*P2X displays an unusual phenotype in that it has a relatively low sensitivity for ATP (EC_50 _~44.5 μM) but has very fast activation and desensitisation kinetics. This is interesting from a mechanistic point of view regarding the relationship between channel gating and agonist binding since it demonstrates that that fast channel kinetics do not necessarily have to be associated with a high agonist sensitivity. Similar to *Hd*P2X, a P2X_3_-like receptor from Zebrafish has also been shown to combine fast current kinetics with low sensitivity to ATP [33]. However, unlike *Hd*P2X (Fig. 3A) current amplitudes at the Zebrafish channel never fully recover from the first stimulation. Other lower organism P2X receptors that have been characterised to date have all displayed slow rates of desensitisation making *Hd*P2X the first example of a non vertebrate P2X channel with fast kinetics. Given that it is likely that a fast phenotype evolved independently in P2X_1,3 _and *Hd*P2X channels, it will be interesting to determine whether the same factors determine the rates of current activation and desensitisation in these evolutionary remote channels.

*Hd*P2X contains several amino acid residues that are highly conserved throughout the vertebrate P2X receptor family. The ten conserved cysteine residues present in the extracellular loop of all vertebrate P2X receptors are thought to form five disulphide bonds [2] and these residues are also present in *Hd*P2X suggesting conservation of gross extracellular structure constrained by disulphide bond location. It is interesting to note that the P2X receptors from *O. tauri *and *M. brevicollis *[11] possess only eight of these ten conserved cysteine residues in their putative extracellular regions and that in each case the two absent cysteines correspond to a proposed disulphide bond pairing in the human P2X_1 _receptor [2] (the equivalents of hP2X_1 _C217 and C227 are absent in *O. tauri *and C117 and C165 are absent in *M. brevicollis*). The agonist binding site of P2X receptors is distinct from other ATP-binding proteins and is thought to be formed from the interaction between adjacent subunits [34]. Based on site directed mutagenesis combined with methanethiosulfonate reactivity a core common mode of action of ATP binding at P2X receptors has been proposed consisting of lysine residues adjacent to the transmembrane domains coordinating phosphate binding, an NFR motif binding the adenine/ribose moiety and an FT motif also involved in agonist action [21]. The lysine residues, NFR and FT motifs are also present in *Hd*P2X (Fig. 1) suggesting that this mechanism of ATP binding arose relatively early in evolution and is not restricted to mammalian receptors. However, the NFR motif and the equivalent of hP2X_1 _K68 are absent in the *D. discoideum *P2XA receptor [10], the FT motif is YT in *S. mansoni *P2X [12] and the NFR motif is NIR in the *O. tauri *[11] receptor suggesting that conservation of the residues involved in ATP binding is not universal to all P2X receptors. Residues at positions D280 and R278 in the rat P2X_4 _receptor have also been shown to play an important role in the action of ATP [35]. These residues are also conserved in *Hd*P2X, *S. mansoni *P2X and *M. brevicollis *P2X suggesting that, despite their incomplete conservation amongst the seven mammalian P2X subtypes, these two residues may also play an important role in the formation of the ATP binding site in some P2X receptor subtypes. Interestingly, the only feature with complete conservation across all vertebrate and lower organism P2X receptors characterised to date is a consensus protein kinase C phosphorylation site in the N-terminal tail of the receptor (Fig. 1). This putative phosphorylation site has been shown to regulate desensitisation in both P2X_1 _[36] and P2X_2 _[20] receptors however, surprisingly direct phosphorylation of this site has been shown not to occur [37,38].

Unlike the *D. discoideum *[10] and *O. tauri *[11] P2X receptors, the general P2 receptor antagonists suramin and PPADS both inhibited *Hd*P2X receptor currents in a concentration dependent manner. PPADS at 100 μM effectively blocked ATP evoked currents. Suramin however was less potent and a component of the current was resistant to antagonism by suramin even at high concentrations. At the highest concentration tested (300 μM) only ~75% of the 100 μM ATP-evoked current was blocked. These properties are opposite to those of the *S. mansoni *P2X, which was more potently inhibited by suramin and incompletely blocked by PPADS [12]. The presence of a lysine residue at position 246 in rat P2X_2 _and at equivalent positions in other mammalian subunits has been proposed to be involved in the inhibition of P2X receptors by PPADS, possibly forming a Schiff base with the aldehyde group of PPADS [39]. Similarly a lysine residue at position 138 in human P2X_1 _has recently been shown to play a key role in the binding of suramin [40]. Neither of these lysine residues shown to be involved in PPADS and suramin action at mammalian receptors are conserved in either *Hd*P2X or *S. mansoni *P2X suggesting that additional residues in the ectodomain also play key roles in the mechanism of antagonist binding.

*Hd*P2X is inhibited by both copper and zinc in a concentration dependent manner (Fig. 5). The effects of these metal ions on vertebrate P2X receptors differ markedly with P2X_1 _being inhibited by zinc, P2X_7 _inhibited by both zinc and copper, P2X_2 _potentiated by both zinc and copper and P2X_4 _potentiated by zinc but inhibited by copper (reviewed by [41]). The action of these metal ions at P2X receptors has physiological significance since both zinc and copper are stored in presynaptic terminals and released after nerve stimulation [41,42]. In addition to *Hd*P2X, the modulation of P2X receptors by metal ions is also present in other lower organisms with *Dictyostelium *P2X inhibited by copper [10] and *S. mansoni *P2X inhibited by zinc [27]. Several mutagenic studies have identified amino acid residues involved in metal binding in P2X_2, 4 and 7 _receptors (reviewed by [41]) however, no consensus binding site has emerged showing independent evolution of metal binding sites among the different vertebrate P2X receptor subtypes. Histidine residues appear to be a common feature in metal binding sites in P2X_2, 4 and 7 _receptors (reviewed by[41]) and in rat P2X_2_, H120 and H213 have been shown to form an intersubunit zinc binding site [26]. There are three extracellular histidine residues present in *Hd*P2X and we mutated each of these residues, both individually and in combination with each other, to alanine in order to assess their involvement in metal binding. Surprisingly, none of the extracellular *Hd*P2X histidine residues are essential for inhibition by zinc or copper (Fig. 5B). This demonstrates that the mechanisms by which metal ions can influence the properties of P2X receptor function are not restricted to interactions with histidine residues. However, mutation of H306 in combination with either H252 or H232 did produce a significant reduction in copper but not zinc inhibition. This suggests that H306 plays a minor but non critical role in the formation of the copper binding site and that the copper and zinc binding sites in *Hd*P2X are distinct from one another. Distinct zinc and copper binding sites are also likely for P2X_4 _which is potentiated by zinc but inhibited by copper [43].

Of the currently identified vertebrate and lower organism P2X receptors only human and rat P2X_4 _[29] and *S. mansoni *P2X [12] are known to be potentiated by the semi synthetic macrocyclic lactone ivermectin. Both P2X_4 _and *S. mansoni *P2X have a slow current phenotype and it is therefore interesting with respect to the mechanism of ivermectin action that *Hd*P2X which has very fast current activation and desensitisation kinetics is also potentiated (Fig. 6). Ivermectin does not affect the kinetics of desensitization of *Hd*P2X currents, a feature also observed in *S. mansoni *P2X. However, in human P2X_4_, ivermectin confers a slower rate of desensitization [29]. Ivermectin is thought to have an allosteric mode of action by increasing maximum current when binding to a high affinity site on the receptor to stabilise the open state, whereas binding to a low affinity site is proposed to slow the rate of deactivation [44]. More recently it was proposed that during gating TM1 rotates relative to TM2, and ivermectin is able to access the lipid environment and optimally bind to a hydrophobic crevice created by TM1 and TM2 at the protein-lipid interface [45]. Interestingly several hydrophobic-nonpolar residues in both TM1 and TM2 that have been shown to be involved in the action of ivermectin at P2X_4 _are conserved in *S. mansoni *P2X [28] and these hydrophobic-nonpolar residues are mostly also present *Hd*P2X. Future investigations comparing the mode of ivermectin action in both naturally fast and slow desensitising P2X channels may enhance our understanding of the process of gating and motions of the channel pore.

## Conclusion

The identification and pharmacological characterisation of a P2X receptor from *H. dujardini *provides further evidence that P2X receptors for ATP emerged early in eukaryotic evolution. Several unusual characteristics of *Hd*P2X including fast current kinetics with low ATP sensitivity, ivermectin potentiation in a channel with fast kinetics and the likelihood of distinct copper and zinc metal binding sites make this channel a useful model for comparative structure-function studies allowing a better understanding of P2X receptors in higher organisms.

## Methods

### Identification of the Hypsibius dujardini P2X receptor

BLAST searches of the GenBank EST database identified a partial *Hypsibius dujardini *EST sequence [GenBank:CO741227] encoding a peptide with significant sequence similarity to the amino terminus of vertebrate P2X receptors. The insert of the corresponding clone for this EST sequence (Hd_mx23_13F10) was fully sequenced on both strands (Automated ABI sequencing service, Leicester University, U.K.) using vector (pSPORT1) and insert specific primers and was shown to contain a full length coding sequence which was named *Hd*P2X.

### Phylogenetic analysis

P2X protein sequences were obtained from SwissProt/UniProt and aligned with ClustalW. The alignment was adjusted by eye to minimise unique indels and subjected to phylogenetic analysis in PAUP* version 4.10b using Neighbour Joining (BioNJ) and maximum parsimony methods. The alignment was 696 characters long, and had 147 invariant and 433 parsimony-informative characters. As the N-terminal and C-terminal portions of the proteins were poorly aligned, the analysis considered only the central 445 characters (of which 342 were parsimony informative, and 208 included gaps). The alignment is available from the authors (MLB). Support for the derived phylogenies was assessed by 1000 bootstrap iterations.

### Site directed mutagenesis

Point mutations in the *Hd*P2X plasmid construct were introduced using the QuikChange™ Mutagenesis Kit (Stratagene, U.S.A.) according to the manufacturer's instructions. Histidines at positions 232, 252 and 306, glutamic acid residues at positions 110 and 249, an aspartic acid residue at position 183 and lysine residues at positions 130 and 297 were mutated to alanine. Double mutations where two of the three histidines were both mutated to alanine were created by conducting serial rounds of site directed mutagenesis as follows: H252A/H232A, H252A/H306A, H232A/H306A. A triple mutant where all three histidines were mutated to alanine was constructed by introducing the H306A mutation into the H252A/H232A double mutant. In all mutants, introduction of the correct mutation(s) and the absence of spontaneous mutations were confirmed by DNA sequencing (Automated ABI sequencing service, Leicester University).

### Oocyte preparation

Mutant and wild type plasmids were digested with *NotI *to linearise and sense strand cRNA was generated using the T7 mMessage mMachine™ kit (Ambion, U.S.A.) according to the manufacturer's instructions. Manually defolliculated stage V-VI *Xenopus *oocytes were injected with 5 ng of cRNA (50 nl at a concentration of 0.1 μg/μl) using an Inject +Matic micro injector (J. Alejandro Gaby, Genève) and were stored at 18°C in ND96 buffer (96 mM NaCl, 2 mM KCl, 1.8 mM CaCl_2_, 1 mM MgCl_2_, 5 mM sodium pyruvate, and 5 mM HEPES, pH 7.5) before electrophysiological recordings were carried out 3–6 days later.

### Two-electrode voltage clamp

Two-electrode voltage clamp recordings were made from oocytes using a Turbo TEC 10C amplifier (NPI Electronic Instruments, Germany) with a Digidata 1200 analogue to digital converter (Axon Instruments U.S.A.) and WinWCP acquisition software (Dr J. Dempster University of Strathclyde, Scotland). Microelectrodes were filled with 3 M KCl, and the external solution consisted of ND96 buffer with 1.8 mM CaCl_2 _replaced with 1.8 mM BaCl^2 ^to prevent the activation of endogenous calcium-activated chloride channels. Membrane currents were recorded at a holding potential of -60 mV. Agonists, ATP (Mg^2+ ^salt), 2',3'-O-4-Benzoylbenzoyl ATP (Bz-ATP) and α,β-methylene-adenosine 5'-triphosphate (αβmeATP) (Sigma, Poole, U.K.) were applied from a U-tube perfusion system, whereas ivermectin, pyridoxal-phosphate-6-azophenyl-2',4'-disulfonic acid (PPADS), suramin, zinc, copper and altered pH solutions were bath-perfused as well as being present at the same concentration in the U-tube solution together with the agonist. Concentration-response curves were constructed by having a 5-minute recovery period between applications and by normalizing data points to two applications (one preceding and one following the test agonist/antagonist concentration). 100 μM ATP was used for normalisation as this concentration produced reproducible responses with a 5 minute recovery interval.

### Data analysis

Data are presented as means ± S.E. Differences between means were assessed by either the Student's t-test for simple comparison between two data sets or by ANOVA followed by Dunnett's post test when multiple data sets were compared (GraphPad Prism software (La Jolla, USA)). Concentration-response data were fitted with the equation *Y *= ((*X*)^*H*^·*M*)/((*X*)^*H *^+ (EC_50_)^*H*^), where *Y *is response, *X *is agonist concentration, *H *is the Hill coefficient, *M *is maximum response, and EC_50 _is the concentration of agonist evoking 50% of the maximum response. pEC_50 _is the -log_10 _of the EC_50 _value. All concentration-response curves, EC_50 _values and Hill coefficients were obtained using GraphPad Prism software.

## Abbreviations

PPADS: Pyridoxal-phosphate-6-azophenyl-2',4' disulfonic acid; αβmeATP: α,β-methylene-adenosine 5'-triphosphate; ADP_hex_: Hexokinase treated ADP.

## Authors' contributions

SB carried out electrophysiological recordings, wrote the first draft of the manuscript and analysed the data. VAS participated in the design of the study and helped draft the manuscript. MLB conducted the phylogenetic analysis, helped draft the manuscript and generated the *Hd*P2X EST clone. SJE conceived and designed the study, performed the molecular biological aspects of the study and wrote the manuscript. All authors read and approved the final manuscript.
